# *Adcyap1* polymorphism covaries with breeding latitude in a Nearctic migratory songbird, the Wilson's warbler (*Cardellina pusilla*)

**DOI:** 10.1002/ece3.2053

**Published:** 2016-04-07

**Authors:** Gaia Bazzi, Andrea Galimberti, Quentin R. Hays, Ilaria Bruni, Jacopo G. Cecere, Luca Gianfranceschi, Keith A. Hobson, Yolanda E. Morbey, Nicola Saino, Christopher G. Guglielmo, Diego Rubolini

**Affiliations:** ^1^ Dipartimento di BioscienzeUniversità degli Studi di Milanovia Celoria 26I‐20133MilanItaly; ^2^ ZooPlantLabDipartimento di Biotecnologie e BioscienzeUniversità degli Studi di Milano‐BicoccaPiazza della Scienza 2I‐20126MilanItaly; ^3^ Department of BiologyAdvanced Facility for Avian ResearchUniversity of Western OntarioLondonOntarioN6A 5B7Canada; ^4^ Natural Resources DepartmentEastern New Mexico University ‐ RuidosoRuidosoNew Mexico88345; ^5^ ISPRA – Istituto Superiore per la Protezione e la Ricerca AmbientaleVia Cà Fornacetta 9I‐40064Ozzano dell'Emilia (BO)Italy; ^6^ Environment Canada11 Innovation BoulevardSaskatoonSaskatchewanS7N 3H5Canada

**Keywords:** Candidate genes, *Clock*, deuterium, migration distance, phenology, stable isotopes

## Abstract

Understanding the genetic background of complex behavioral traits, showing multigenic control and extensive environmental effects, is a challenging task. Among such traits, migration is known to show a large additive genetic component. Yet, the identification of specific genes or gene regions explaining phenotypic variance in migratory behavior has received less attention. Migration ultimately depends on seasonal cycles, and polymorphism at phenological candidate genes may underlie variation in timing of migration or other aspects of migratory behavior. In this study of a Nearctic–Neotropical migratory songbird, the Wilson's warbler (*Cardellina pusilla*), we investigated the association between polymorphism at two phenological candidate genes, *Clock* and *Adcyap1*, and two aspects of the migratory phenotype, timing of spring migration through a stopover site and inferred latitude of the breeding destination. The breeding destination of migrating individuals was identified using feather deuterium ratio (*δ* ^2^H), which reliably reflects breeding latitude throughout the species' western breeding range. Ninety‐eight percent of the individuals were homozygous at *Clock*, and the rare heterozygotes did not deviate from homozygous migration phenology. *Adcyap1* was highly polymorphic, and allele size was not significantly associated with migration date. However, *Adcyap1* allele size significantly positively predicted the inferred breeding latitude of males but not of females. Moreover, we found a strong positive association between inferred breeding latitude and *Adcyap1* allele size in long‐distance migrating birds from the northern sector of the breeding range (western Canada), while this was not the case in short‐distance migrating birds from the southern sector of the breeding range (coastal California). Our findings support previous evidence for a role of *Adcyap1* in shaping the avian migratory phenotype, while highlighting that patterns of phenological candidate gene–phenotype associations may be complex, significantly varying between geographically distinct populations and even between the sexes.

## Introduction

In many migratory organisms, individuals embark on their first migratory journey without any guidance from conspecifics concerning the timing and the direction of migration and the distance to be covered, and in most cases in the absence of any cue concerning the ecological conditions they subsequently face (Newton 2008). Such an innate spatiotemporal program, which is remarkably consistent at the population level, hints at a strong genetic component of migratory behavior (reviewed in Liedvogel and Lundberg 2014). Indeed, experimental studies of birds focusing on quantitative genetics and heritability of timing and direction of migration and migration distance revealed these traits have a large additive genetic component (Pulido and Berthold 2003). However, identifying which individual genes or gene groups are involved in shaping phenotypic variability in natural populations of migratory species has received less attention (Liedvogel and Lundberg 2014).

Studies of migratory vertebrates have mainly focused on phenological candidate genes (PCG), that is, genes whose allelic variation may explain differences in the photoperiodic responses among populations and individuals (e.g., Johnsen et al. 2007; O'Malley and Banks 2008; O'Malley et al. 2010; Bourret and Garant 2015; Saino et al. 2015). These PCG show short tandem repeats that could affect gene functions and are possibly involved in determining variability in migratory phenotypes (Kashi et al. 1997; Comings 1998; Li et al. 2004; Fondon et al. 2008). Among PCG, *Clock* (*Circadian Locomotor Output Cycles Kaput*) plays a central role within the “core circadian oscillator” (CCO), which is ultimately responsible for the onset and setting of circadian and circannual rhythmicity (Panda et al. 2002; Lincoln et al. 2003; Bell‐Pedersen et al. 2005; Ko and Takahashi 2006). Polymorphism at a *Clock* polyglutamine‐rich region (Poly‐Q) has been reported both among and within populations (e.g., Johnsen et al. 2007; O'Malley and Banks 2008; Liedvogel et al. 2009; Caprioli et al. 2012), and it may play a role in determining phenological responses by differentially affecting the timing of seasonal activities (Hayasaka et al. 2002). At the among‐population level, a latitudinal cline in *Clock* allele size, with the frequency of longer alleles increasing along a south–north gradient, has been documented in some migratory bird and fish species (Johnsen et al. 2007; O'Malley and Banks 2008; O'Malley et al. 2010; Lemay and Russello 2014) and probably reflects local adaptation to different photoperiodic regimes (Kyriacou et al. 2008). However, such a latitudinal cline has not been detected in other studies (e.g., Dor et al. 2012; O'Brien et al. 2013).

Within populations, *Clock* polymorphism may affect an individual's response to photoperiod. Intrapopulation variation in *Clock* allele size predicted timing of key life‐history events of birds, such as reproduction, molt, and migration (Liedvogel et al. 2009; Caprioli et al. 2012; Saino et al. 2013, 2015; Bazzi et al. 2015; Bourret and Garant 2015), with individuals bearing “shorter” alleles (i.e., with fewer glutamine residues) showing advanced timing, while individuals bearing “longer” alleles showed a delayed phenology. Yet, other studies failed to find any association between *Clock* genotype and timing of phenophases (Liedvogel and Sheldon 2010; Dor et al. 2011; Chakarov et al. 2013; Morbey et al. 2014). Finally, in a Nearctic migratory bird species complex (*Junco hyemalis* and *J. phaeonotus*), *Clock* allele size increased with migration distance within some subspecies groups, but not among populations (Peterson et al. 2013).

A second important PCG, *Adcyap1* (*Adenylate Cyclase‐Activating Polypeptide 1*), encodes PACAP (pituitary adenylate cyclase‐activating polypeptide), a neuropeptide broadly diffused in the brain and peripheral organs of vertebrates (Nowak and Zawilska 2003; Vaudry et al. 2009; Olano‐Marin et al. 2011). PACAP is known to exert several biological functions, many of which could foster physiological and behavioral shifts related to migration (Mueller et al. 2011). PACAP plays a role in circadian and circannual timing, stimulating melatonin synthesis in the pineal gland and conveying light information from the retina to the suprachiasmatic nucleus of the hypothalamus, a key element in the regulation of circadian timing of birds and mammals (Simonneaux et al. 1993; Hannibal et al. 1997; Schwartz and Andrews 2013). PACAP also modulates the expression of the CCO, by directly activating *Clock* and other circadian genes in the chicken pineal gland (Nagy and Csernus 2007; Racz et al. 2008). *Adcyap1* shows microsatellite polymorphism at the 3′‐UTR of the gene that has been suggested to modify post‐transcriptional processes (Steinmeyer et al. 2009). The first evidence of a link between *Adcyap1* 3′‐UTR polymorphism and migratory behavior came from a study of the blackcap (*Sylvia atricapilla*), where longer *Adcyap1* allele sizes were positively associated with the amount of migratory restlessness of caged birds during their first migration toward the wintering areas (Mueller et al. 2011). Hence, allele size variation may modulate migration distance within populations, with birds bearing longer *Adcyap1* alleles migrating farther, because the amount of migratory restlessness is associated with longer migratory flights (Gwinner 1990; Berthold 1996; Maggini and Bairlein 2010). Furthermore, blackcap populations migrating over longer distances had a longer mean *Adcyap1* allele size (Mueller et al. 2011). A study of the dark‐eyed junco (*J. hyemalis*) further supported the possible role of *Adcyap1* in affecting migratory restlessness in a migratory population (Peterson et al. 2013). However, *Adcyap1* polymorphism was not associated with migration distance among junco populations (Peterson et al. 2013).

As PACAP modulates molecular clocks (Nagy and Csernus 2007; Racz et al. 2008), we may also expect *Adcyap1* to predict phenology within populations. Early dispersing juvenile buzzards (*Buteo buteo*) carried longer *Adcyap1* alleles (Chakarov et al. 2013). Moreover, *Adcyap1* allele size predicted laying date of female tree swallows (*Tachycineta bicolor*), although the effect of genotype on phenology varied between local populations (Bourret and Garant 2015). Finally, there was no association between timing of spring migration toward the breeding areas and *Adcyap1* polymorphism in four Palearctic–Afrotropical migrants (Saino et al. 2015), and *Adcyap1* polymorphism predicted timing of spring migration in blackcaps migrating across Europe only among females and in combination with wing morphology (Mettler et al. 2015).

**Figure 1:**
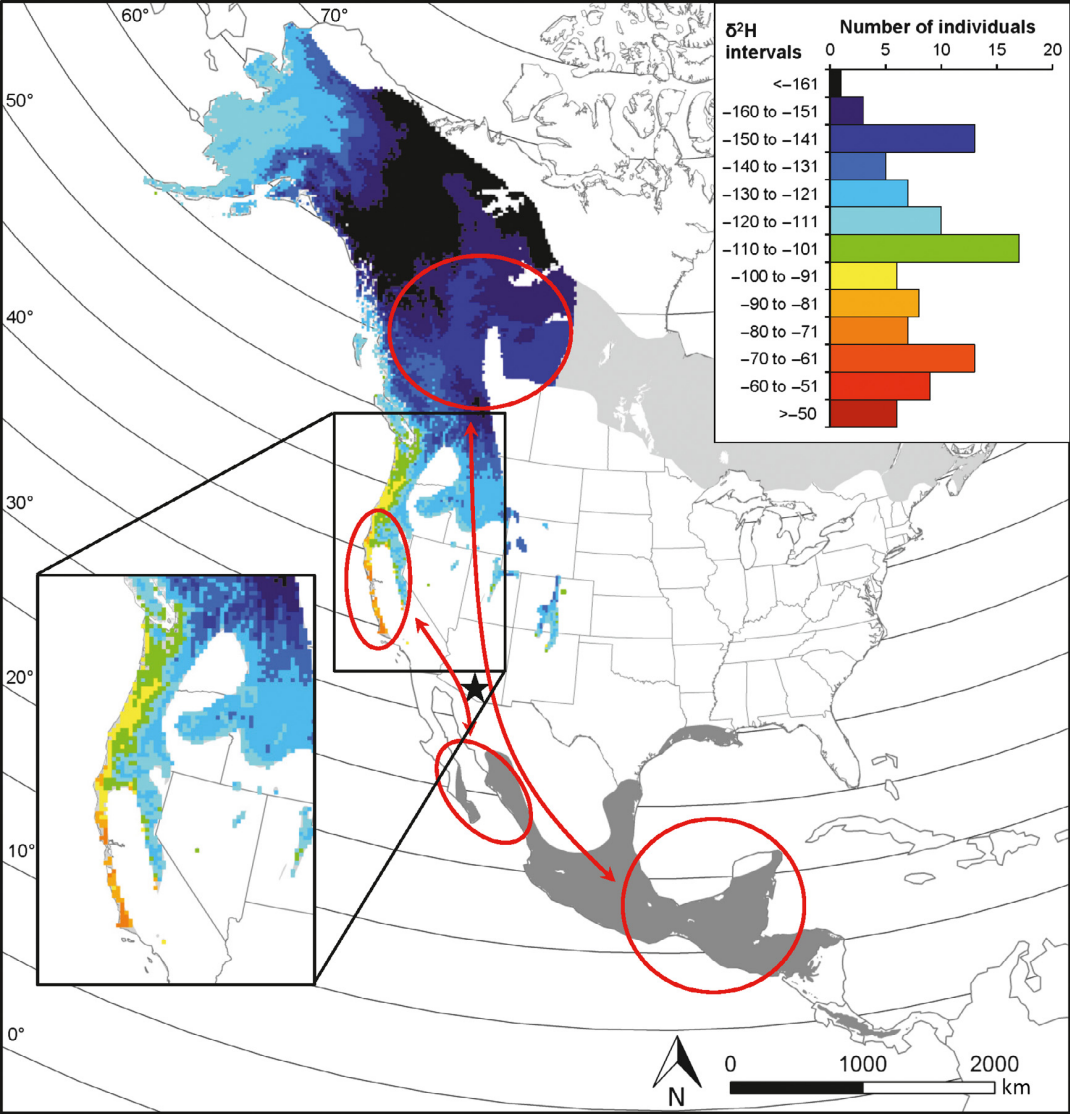
Feather *δ* ^2^H isoscape within the breeding range of western North American Wilson's warbler populations. The map (Lambert Conformal Conic projection) shows the expected spatial distribution of feather *δ* ^2^H values in the western part of Wilson's warbler breeding range (see Materials and Methods). The eastern portion of the breeding distribution range (light gray) and the entire wintering distribution range (dark gray) of the species is also shown for comparison, and the position of the study site (Buenos Aires National Wildlife Refuge, Arizona) is marked as a black star. Inset histogram: frequency distribution of feather *δ* ^2^H values of spring migrating Wilson's warblers at the study site (*n = *105). The ellipses denote the approximate breeding destination (based on feather *δ* ^2^H thresholds; see Materials and Methods) and wintering distribution (based on Ruegg et al. 2014b) of short‐distance migrating southern birds (feather *δ* ^2^H ≥ −90‰, *n = *43 individuals) and of long‐distance migrating northern birds (feather *δ* ^2^H values < −130‰, *n = *22 individuals) (see Materials and Methods).

In this study of a Nearctic–Neotropical migratory passerine, the Wilson's warbler (*Cardellina pusilla*), we aimed to assess whether *Clock* and *Adcyap1* polymorphism predicted the timing of spring migration to breeding areas, recorded at a southern stopover site, and the inferred latitude of the breeding destination, as gauged from the stable hydrogen isotope ratio of feathers (Kelly et al. 2002; Paxton et al. 2007; Hobson and Wassenaar 2008). We sampled Wilson's warblers during spring migration along the western flyway at a stopover site in Arizona, USA, halfway between the wintering (from southern Mexico to Panama) and the breeding areas (from California to northern Canada and Alaska) (see Fig. 1). The Wilson's warbler is an obligate migrant which follows a “leapfrog” migration pattern, with birds from northern breeding populations overwintering at more southern latitudes in the wintering range (Central Mexico to Panama) compared to those from southern breeding populations, that overwinter in northern Mexico (Kelly et al. 2002; Clegg et al. 2003; Paxton et al. 2007; Ruegg et al. 2014b). Hence, birds from northern breeding populations perform considerably longer migrations than those from southern breeding ones (Fig. 1).

We took advantage of the well‐defined geographical gradient in hydrogen stable isotope ratios (*δ* ^2^H hereafter) in precipitation across North America to infer the breeding destination of migrating warblers sampled at the study site (Fig. 1; Kelly et al. 2002; Clegg et al. 2003; Paxton et al. 2007; Hobson and Wassenaar 2008; Hobson et al. 2014).

As outlined above, candidate genes may predict phenotypic variation at two different levels: within and among populations. To investigate within‐population effects, we tested the association between timing of migration and genetic polymorphism while statistically removing the confounding effects of breeding latitude (reflecting population of origin; northern populations migrate later, this study and Paxton et al. 2007). On the other hand, to investigate among‐population effects and highlight possible adaptive geographic clines in allele frequencies, we analyzed the association between genetic polymorphism and inferred breeding latitude.

In line with previous literature documenting associations between *Clock* polymorphism and phenology, we predicted that within populations (i.e., after controlling for breeding latitude, by including feather *δ* ^2^H in the models), individuals bearing shorter *Clock* alleles will migrate early, whereas those with longer alleles will have a delayed migration phenology. Among populations, we predicted birds originating from northern populations to have longer *Clock* alleles than those from southern populations.

On the other hand, we expected *Adcyap1* allele size to increase with latitude of the breeding destination (i.e., decrease with increasing feather *δ* ^2^H; Fig. 1), reflecting an among‐population effect. We also explored the association between *Adcyap1* and the timing of migration at the within‐population level (i.e., after controlling for feather *δ* ^2^H), but no clear prediction could be formulated due to the paucity of previous studies (Chakarov et al. 2013; Bourret and Garant 2015; Mettler et al. 2015; Saino et al. 2015).

## Materials and Methods

### Study species

The Wilson's warbler is a small, sexually dichromatic, widespread Nearctic passerine that breeds in North America and winters in Mexico and Central America (Pyle et al. 1997). The species shows a clear genetic differentiation among geographical populations, with birds from the eastern portion of the breeding range and migrating through eastern North America being well separated from those migrating along the western flyway (Ruegg et al. 2014b). In addition, western breeding populations can be separated into five homogeneous genetic clusters (Ruegg et al. 2014b). Wilson's warblers perform a single complete postbreeding molt while at or near the breeding grounds, before fall migration (Pyle et al. 1997; Paxton et al. 2007). Hence, birds sampled during spring migration will carry tail feathers grown in the breeding areas in the previous breeding season. Feather *δ* ^2^H values from spring migrating birds was thus used to infer individual breeding destinations (see below).

### Study site and field methods

We captured Wilson's warblers at the Buenos Aires National Wildlife Refuge (Fig. 1), Pima County, USA (31°33′00″N 111°33′02″W), during March 28–May 24, 2006. All field procedures were approved by the University of Western Ontario Animal Use Sub‐Committee (2006‐014‐02), the US Geological Survey Bird Banding Lab (23423), the US Fish and Wildlife Service (MB121152‐0), and the Arizona Game and Fish Department (298399). The study area, located in the Sonoran Desert, provides isolated riparian habitats to a large number of migrating birds which stopover there to refuel during spring migration. Previous studies showed that birds with markedly different breeding destinations (exclusively from the western breeding range) use the area as a stopover site (Paxton et al. 2007; Ruegg et al. 2014b; Fig. 1). We selected a small (approx. 2 ha), isolated trapping site which enabled us to reliably capture most of the foraging individuals at any given time; afternoon resighting efforts indicated that unmarked individuals were rare (QRH, unpublished data). We are therefore confident that almost all the birds were captured on the day of arrival. We used first capture date of each bird as a reliable estimate of timing of migration (hereafter, migration date).

Birds were trapped during the morning hours using mist nets, banded, measured (wing length, to the nearest mm), and sexed according to Pyle et al. (1997). Age was not assessed due to difficulties in discriminating between second year and older individuals based on the differences in feather wear. For each individual, we collected a biological sample including one outer rectrix and three breast contour feathers. Full‐grown feathers were collected and stored individually at room temperature for later analyses. Overall, we captured 108 individuals (49 females, 59 males).

### Stable isotope analyses and feather isoscape of the breeding destination

Stable hydrogen isotope measurements were made for individual rectrices. *δ* ^2^H analyses were performed using the comparative equilibration approach reported in Wassenaar and Hobson (2003) (see Appendix S1 for details). Isotope values were reported in *δ* notation as parts per thousand (‰) deviation from the Vienna Standard Mean Ocean Water (VSMOW)–Standard Light Antarctic Precipitation (SLAP) scale. We could determine *δ* ^2^H for 105 individuals (47 females, 58 males) of the 108 sampled. The remains of analyzed feathers were kept in glass tubes at room temperature until genetic analyses were performed.

To infer the putative breeding destination of migrating Wilson's warblers, we compared the feather *δ* ^2^H values with a *δ* ^2^H feather isoscape of North America showing the spatial distribution of expected feather *δ* ^2^H values on a continental scale (Fig. 1). The *δ* ^2^H feather isoscape was derived by combining the most recent available amount‐weighted growing season *δ* ^2^H precipitation surface (see Terzer et al. 2013 for details and data source) and a feather – precipitation *δ* ^2^H transfer function for Neotropical migrant nonground foragers (full details reported in Hobson et al. 2014).

To compare the strength of genotype–phenotype associations between populations differing in migratory behavior (see Within‐population variation in timing of migration and genotype below), we aimed at separating long‐distance migrating “northern” birds from short‐distance migrating “southern” ones. Moreover, in these comparisons, we aimed at accounting for the geographical genetic structuring among the species' western population complex previously described by Ruegg et al. (2014b) using high‐resolution genetic markers. To this end, we first assigned our feather *δ* ^2^H values to 10‰ intervals and plotted the contours of these intervals on the feather isoscape map (Fig. 1). “Southern” birds were identified as those with feather *δ* ^2^H values ≥ −90‰ (*n = *43 individuals), which is consistent with the genetically homogeneous cluster of coastal California breeding birds, migrating south over short‐distances to the Pacific coastal areas of northern Mexico (“yellow” cluster in Ruegg et al. 2014b; Fig. 1). “Northern” birds were identified as those with feather *δ* ^2^H values < −130‰ (*n = *22 individuals), which breed throughout Canada and migrate over long distances to winter in southern Mexico and throughout Central America (Kelly et al. 2002; Ruegg et al. 2014b; Fig. 1). These birds belong to a different genetically homogeneous cluster (“violet” cluster in Ruegg et al. 2014b). Intermediate feather *δ* ^2^H values (between −130‰ and −91‰) were excluded from these analyses as they may either reflect birds breeding in continental northwestern US states or in central‐western Alaska (see Fig. 1), thus including birds from genetically differentiated clusters of populations (Ruegg et al. 2014b). Nevertheless, given that the mean value of the lowest 10^th^ percentile of the feather *δ* ^2^H values we recorded in migrating individuals (−150.7‰; see also Fig. 1) corresponds to ca. 59° N on the feather isoscape shown in Fig. 1 (details not shown), we regard the possibility that birds from northern and northwestern Canada and Alaska (i.e., above 60° N, Fig. 1) were included in our sample as unlikely. Indeed, these populations may show a more eastward migration route (Paxton et al. 2007). Finally, Kelly et al. (2002) showed that feather *δ* ^2^H of Wilson's warblers sampled throughout their entire western breeding range (from California to northern Alaska) decreases linearly with breeding latitude. Hence, we can safely assume that, in our sample, decreasing feather *δ* ^2^H values reflects increasing latitudes of breeding destinations.

### Genetic analyses

Total genomic DNA was extracted from feathers (both the rectrix remains and breast contour feathers) using commercial kits (see Appendix S1 for details). Polymorphism at the *Clock* and *Adcyap1* genes (allele size due to the number of sequence repeats) was determined by fragment analysis as described in Caprioli et al. (2012) and Saino et al. (2015) with slight modifications (Appendix S1). Of the 105 birds with feather *δ* ^2^H values, we reliably genotyped 102 individuals (*Clock*: 46 females, 56 males; *Adcyap1*: 44 females, 58 males). Although no *Clock* genomic sequence is available for the study species, the alignment of all *Clock* gene sequences of passerine species found in GenBank showed that all length polymorphisms in this order are due to a variable number of glutamine codons (details not shown). Hence, it is safe to assume that the 115‐ and 118‐bp‐long alleles we detected (see Results) correspond to Q_8_ and Q_9_ alleles, respectively (*Clock* alleles are identified according the predicted number of glutamine residues in the mature protein; see Liedvogel et al. 2009).

### Statistical analyses

As we found extremely low variability at the *Clock* locus (see Results), to explore the *Clock* genotype‐timing of migration association, we compared migration date of the rare heterozygote *Clock* individuals with the phenotypic distribution of migration date of the homozygote ones (as detailed in Bazzi et al. 2015). We thus calculated the 95% nonparametric bootstrap confidence limits (BCa method; see details in Bazzi et al. 2015) of migration date for homozygous individuals, separately for each sex; to account for seasonal variation in the occurrence of birds from different breeding destinations (see Results and Paxton et al. 2007), the migration date values of both the homozygote and the heterozygote individuals were expressed as residuals from the linear regression of migration date on feather *δ* ^2^H, separately for each sex. We then compared the heterozygote values (residuals) with the confidence intervals of homozygote individuals (from the distribution of residuals) of their respective sex. No formal analysis was made to investigate the association between *Clock* genotype and breeding destination.

To test for an association between *Adcyap1* allele size and migration date while controlling for variation in migration date due to variation in breeding destination, we ran linear models of migration date (1 = January 1) as a function of allele size (mean of the two alleles; Mueller et al. 2011; Chakarov et al. 2013; Peterson et al. 2013; Saino et al. 2015), feather *δ* ^2^H, and sex (0 = females, 1 = males). Finally, we tested the association between *Adcyap1* genotype and breeding destination by linear models of feather *δ* ^2^H as a function of *Adcyap1* allele size and sex. Similar models were run to investigate the association between wing length, another proxy of breeding destination (see Results and Q. R. Hays et al. in prep.), and *Adcyap1* allele size. Two‐way interaction terms were included in all initial linear models and removed *en bloc* if statistically nonsignificant (*P *>* *0.05).

We also tested whether the strength of the *Adcyap1*–feather *δ* ^2^H association varied between the two genetically homogeneous clusters of northern and southern birds by computing the correlation coefficients between *Adcyap1* allele size and feather *δ* ^2^H for northern and southern birds, and applying a test for the difference between two correlation coefficients (Sokal and Rohlf 2009).

All the analyses involving *Adcyap1* were repeated for each allele size (short and long) to investigate whether allelic dominance effects occurred, because this is often the case in phenotypic effects of simple sequence repeats (Ross 2002; Fondon et al. 2008; Liedvogel et al. 2009; Saino et al. 2015).

Deviations from Hardy–Weinberg equilibrium (HWE) were tested for the *Adcyap1* locus using the Markov chain method (Guo and Thompson 1992) implemented in GENEPOP (dememorization* *= 1000, batches = 100, iterations per batch = 1000) to obtain unbiased estimates of the Fisher's exact statistic (Raymond and Rousset 1995). For this locus, we also calculated genetic differentiation by computing the *F*_ST_ estimate between the northern and southern populations by means of Fstat 2.9.3 software (Goudet 2001).

Means and parameter estimates are reported together with their associated standard error, unless stated otherwise.

## Results

### Timing of migration and breeding destination

**Figure 2:**
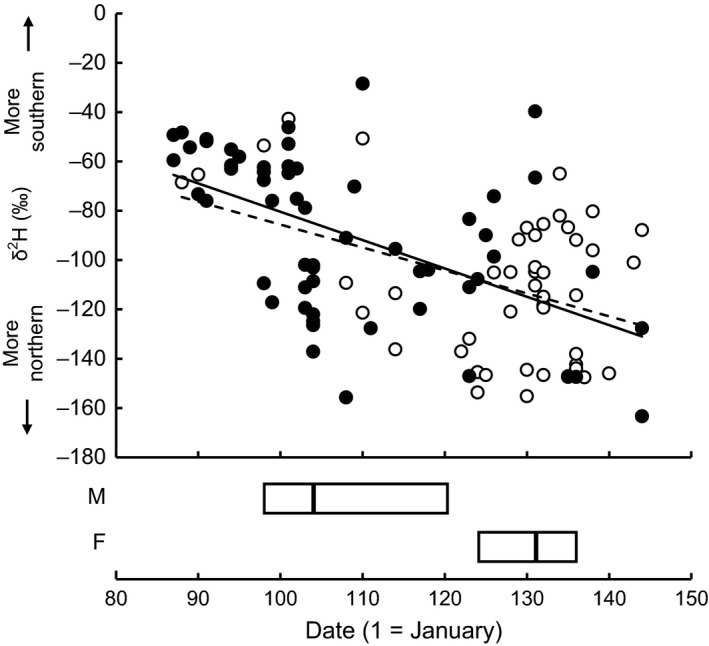
Feather *δ* ^2^H decreases during the migration period in male and female Wilson's warblers. Males: filled circles, continuous line; *n = *58; females: open circles, broken line; *n = *47. The lines represent simple linear regressions. Boxplots show the median migration date of each sex (boxes: 25^th^ and 75^th^ percentile).

Feather *δ* ^2^H values showed a clear temporal decline during the migration period [Fig. 2; linear regression, estimate: −1.08 (0.16) ‰/day, *t*_103_ = 6.67, *P *<* *0.001], with birds directed toward northern breeding areas (i.e., with lower feather *δ* ^2^H values) migrating later (see also Paxton et al. 2007). Migration date also significantly differed between the sexes, males migrating considerably earlier on average (April 19) than females (May 7) (*t*_106_ = 6.76, *P *<* *0.001) (Fig. 2) (see Otahal 1995). Feather *δ* ^2^H predicted migration date in a similar way in either sex (see Fig. 2), as indicated by the nonsignificant interaction between sex and feather *δ* ^2^H in a linear model (*F*_1,101_ = 0.72, *P *=* *0.40). This model also confirmed a statistically significant sex difference in migration date [mean estimated difference in migration date between males and females: 14.33 (2.59) days (*F*_1,102_ = 30.68, *P *<* *0.001)] while controlling for the effect of feather *δ* ^2^H [−0.21 (0.04) days/‰, *F*_1,102_ = 29.76, *P *<* *0.001] (see also above and Fig. 2).

### Genetic variation at *Clock* and *Adcyap1*

The *Clock* locus showed very low variability, because only two (one male, one female) of 102 genotyped individuals were heterozygous (Q8/Q9), while all the others were homozygous (Q9/Q9) [mean allele size = 117.97 (0.02) bp; observed heterozygosity *H*_o_ = 0.02]. On the other hand, *Adcyap1* was highly variable (*H*_o_ = 0.78) (Table S1). *Adcyap1* genotype frequencies significantly deviated from HWE (*P = *0.030). However, this was not the case if the test was conducted within southern and northern birds (both *P*‐values > 0.60). This finding corroborates the hypothesis of reduced gene flow between genetically differentiated southern and northern population clusters (Ruegg et al. 2014b). Indeed, *Adcyap1* genotypes showed a statistically significant differentiation between southern and northern birds (*F*_ST_ = 0.045, *P *=* *0.001). The estimated *F*_ST_ for the *Adcyap1* microsatellite was considerably larger than the mean *F*_ST_ obtained from the reanalysis of the allelic panel of neutral microsatellite loci previously published in Clegg et al. (2003) from birds breeding in the corresponding population clusters (courtesy of S. M. Clegg and T. B. Smith) (see Appendix S2).

However, the mean *Adcyap1* allele size did not significantly differ between southern and northern birds [158.26 (0.19) vs. 158.73 (0.37), respectively; *t*_63_ = 1.28, *P *=* *0.21]. Results were qualitatively similar for the short and long allele size (details not shown for brevity).

### Within‐population variation in timing of migration and genotype

Controlling for feather *δ* ^2^H, the migration dates of the heterozygous *Clock* male and female fell within the 95% confidence limit of the sex‐specific distributions of migration date (Table S2). Hence, the rare heterozygous *Clock* individuals were not phenodeviant with respect to migration timing of homozygotes.

There was no statistically significant association between *Adcyap1* allele size and migration date in linear models controlling for sex and feather *δ* ^2^H (Table 1). There were no significant two‐way interactions between sex, allele size, and feather *δ* ^2^H on migration date (Table 1). Hence, the (nonsignificant) effect of *Adcyap1* genotype on timing of migration was similar irrespective of geographical origin (no statistically significant allele size × *δ* ^2^H interaction, Table 1).

**Table 1:** Linear models of the effect of *Adcyap1* allele size on migration date, feather *δ* ^2^H, and wing length. The interaction terms were removed *en bloc* if nonsignificant (*P *>* *0.05), and statistics for main effects refer to models without the interaction term. Results were qualitatively similar if the short or long allele size was considered instead of the mean allele size (details not shown for brevity)

	Estimate (SE)	df	*F*	*P*
Migration date (*n* = 102)				
Allele size	0.34 (0.86)	1, 98	0.16	0.69
Sex	−14.64 (2.67)	1, 98	30.18	<0.001
*δ* ^2^H	−0.21 (0.04)	1, 98	27.93	<0.001
Allele size × sex	0.21 (1.86)	1, 95	0.01	0.91
Allele size × *δ* ^2^H	−0.01 (0.03)	1, 95	0.09	0.77
Sex × *δ* ^2^H	−0.05 (0.08)	1, 95	0.42	0.52
Feather δ^2^H (*n* = 102)				
Allele size	−4.35 (2.11)	1, 99	4.26	0.042
Sex	21.63 (6.35)	1, 99	11.59	0.001
Allele size × sex	−6.22 (4.32)	1, 98	2.07	0.15
Wing length (*n* = 102)				
Allele size	0.28 (0.12)	1, 99	5.60	0.020
Sex	1.49 (0.35)	1, 99	17.74	<0.001
Allele size × sex	0.21 (0.24)	1, 98	0.78	0.38

### Breeding destination and genotype

The two rare heterozygous *Clock* individuals had feather *δ* ^2^H values that were at the extremes of the feather *δ* ^2^H distribution: The male had a value of −63‰, whereas the female had a value of −145‰. Hence, the male belonged to the southern group, while the female to the northern group (see Materials and Methods) (see also Fig. 1).

**Figure 3:**
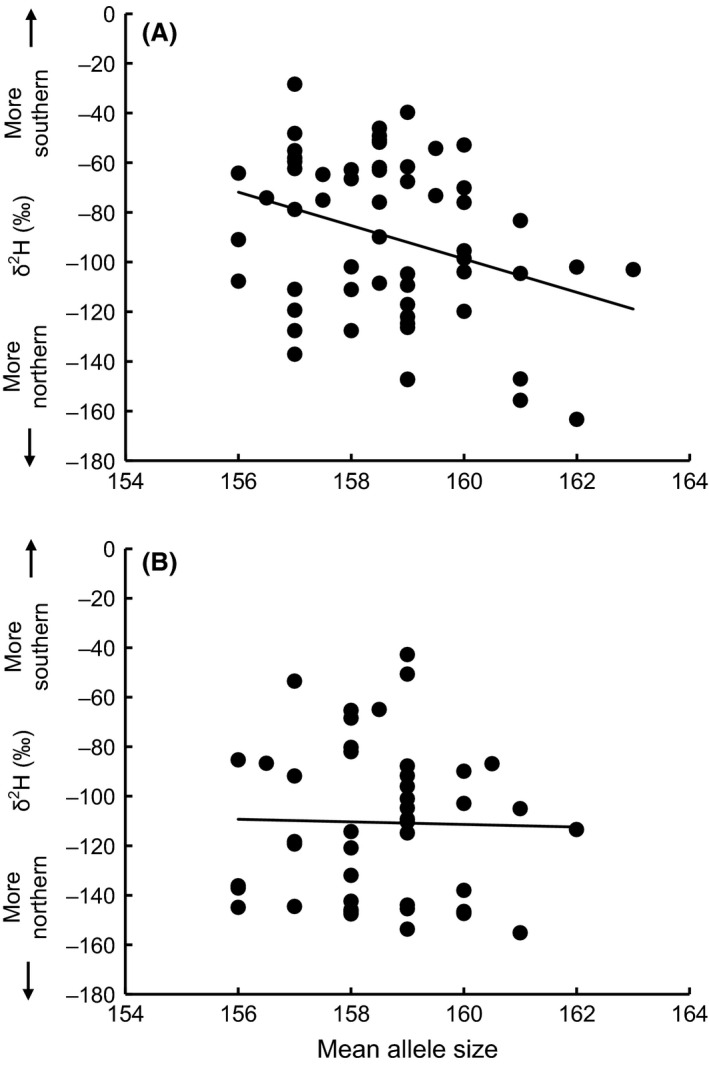
Wilson's warbler feather *δ* ^2^H values in relation to *Adcyap1* allele size in (A) males and (B) females. The lines represent simple linear regressions.

In linear models of feather *δ* ^2^H as a function of mean *Adcyap1* allele size and sex, allele size marginally and negatively predicted feather *δ* ^2^H (*F*_1,99_ = 4.26, *P *=* *0.042; Table 1, Fig. 3). Moreover, females showed significantly lower feather *δ* ^2^H values than males [−110.6‰ (30.2 SD) vs. −89.7‰ (33.0 SD), respectively]. The slope of the relationship between mean allele size and feather *δ* ^2^H was similar in the two sexes, as testified by the nonsignificant interaction between allele size and sex (Table 1). However, the relationship was statistically significant in males (*r *=* *−0.32, *P *=* *0.015, *n = *58) but not in females (*r *=* *−0.02, *P *=* *0.88, *n = *44) (Fig. 3).

The results were similar if the size of the long *Adcyap1* allele was used instead of mean *Adcyap1* allele size (Table 1) (effect of allele size: *F*_1,99_ = 5.15, *P *=* *0.025). The correlation coefficient (Pearson's *r*) between the long allele size and feather *δ* ^2^H in males was ‐0.38 (*P *=* *0.004), whereas in females it was 0.01 (*P *=* *0.97). No statistically significant association emerged between short *Adcyap1* allele size and feather *δ* ^2^H (correlation coefficients: males, *r *=* *−0.14, *P *=* *0.30; females, *r *=* *−0.05, *P *=* *0.74).

Wing length decreased linearly with feather *δ* ^2^H, indicating that wing length can be used as a reliable proxy of breeding destination and that more negative feather *δ* ^2^H values within the observed range of variation were reflecting northern breeding destinations (see also *Stable isotope analyses and feather isoscape of the breeding destination*) [linear model with sex and feather *δ* ^2^H as predictors, effect of feather *δ* ^2^H, estimate: −0.03 (0.01), *F*_1,102_ = 56.63, *P *<* *0.001; effect of sex: *F*_1,102_ = 55.92, *P *<* *0.001; the sex × *δ* ^2^H interaction and the quadratic term of feather *δ* ^2^H were not significant (details not shown)] (see also Q. R. Hays et al., in prep.).

Wing length was significantly (positively) predicted by *Adcyap1* mean and long allele size, while controlling for sex differences in wing length (Table 1; effect of long allele size, estimate: 0.20 (0.09), *F*_1,99_ = 5.27, *P *=* *0.024). The correlation between wing length and mean or long *Adcyap1* allele size was statistically significant in males (mean allele size: *r *=* *0.30, *P *=* *0.021; long allele size: *r *=* *0.33, *P *=* *0.011) but not in females (mean allele size: *r *=* *0.12, *P *=* *0.44; long allele size: *r *=* *0.06, *P *=* *0.68). No statistically significant association emerged between wing length and short *Adcyap1* allele size (effect of short allele size: *F*_1,99_ = 2.48, *P *=* *0.119; correlation coefficients: males, *r *=* *0.14, *P *=* *0.35; females, *r *=* *0.17, *P *=* *0.22). However, controlling for breeding destination, *Adcyap1* allele size (mean, long or short) did not significantly predict wing length anymore (all *P *>* *0.17); hence, the association between wing length and *Adcyap1* genotype was a spurious effect of the latitudinal variation of wing length.

**Figure 4:**
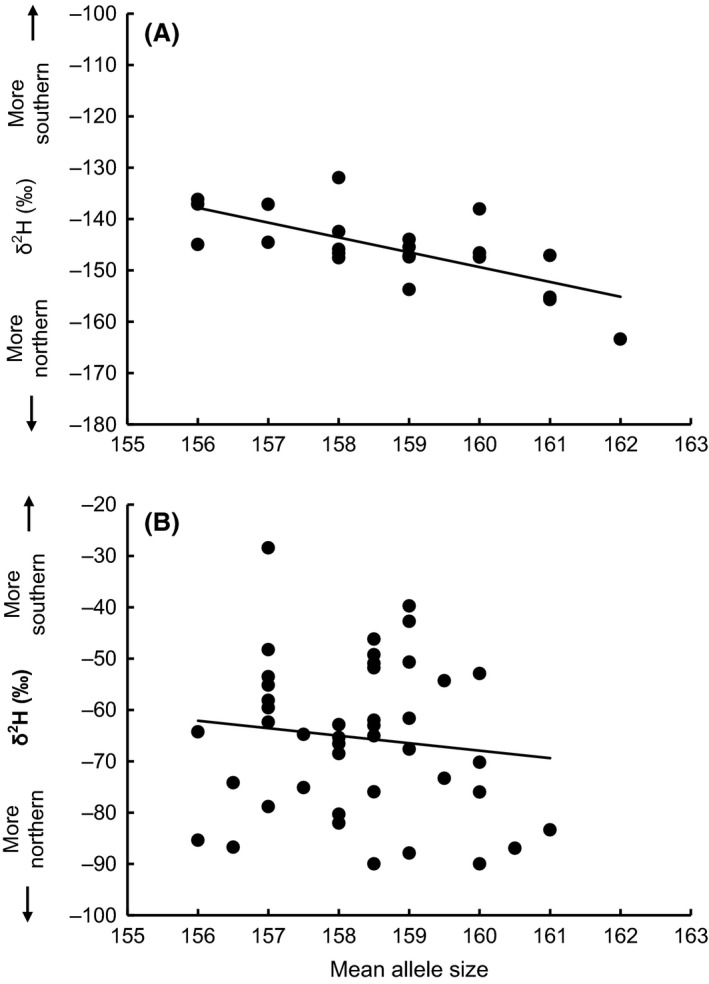
Correlation between *Adcyap1* allele size and feather *δ* ^2^H values in (A) northern or (B) southern birds. The same *y*‐axis range is shown for ease of comparison. The lines represent simple linear regressions.

The strength of the association between *Adcyap1* mean or long (but not short) allele size and feather *δ* ^2^H, as gauged by correlation coefficients, significantly differed between the genetically homogeneous clusters of southern and northern birds (Table 2): *Adcyap1* allele size and feather *δ* ^2^H were strongly negatively correlated among northern birds, while this was not the case among southern birds (Table 2, Fig. 4). Results were qualitatively unaltered if these comparisons were based on more restrictive cutoffs for northern (i.e., < −140‰) and southern (≥ −80‰) birds (Fig. 1 and Table S3).

**Table 2:** Association between *Adcyap1* allele size and feather *δ* ^2^H in southern and northern Wilson's warblers. *Z*‐values refer to the test of the difference between the correlation coefficients of southern and northern birds

	Southern birds (*n = *43)	Northern birds (*n = *22)	*Z*	*P*
Mean allele size	−0.12	−0.69***	2.63	0.009
Short allele size	−0.13	−0.31	0.69	0.490
Long allele size	−0.07	−0.77***	3.44	<0.001

Significance levels of correlation coefficients: ***P <* *0.001.

To further rule out that the strong association between inferred breeding latitude and *Adcyap1* allele size among northern birds is confounded by geographical variation in population genetic structure, we reanalyzed previously published data (Clegg et al. 2003) on (presumably) neutral microsatellite loci from Wilson's warbler breeding in range of northern birds (Table S4). We found no statistically significant association between mean allele size of 8 microsatellite markers and either breeding latitude or predicted feather *δ* ^2^H values at the breeding sites (Table S4).

## Discussion

We investigated whether variation at two PCG, *Clock* and *Adcyap1*, affected the timing of spring migration and breeding destination, as inferred from feather *δ* ^2^H values. To our knowledge, this is the first study testing whether interindividual variation in migration distance was predicted by *Clock* and *Adcyap1* genetic polymorphism. We found low variability at the *Clock* gene locus, most individuals being homozygous, and the two rare *Clock* heterozygotes were not deviants compared to homozygotes for timing of migration. Hence, rare genetic variants of this *Clock* gene region are not associated with migratory behavior in Wilson's warbler.

The *Adcyap1* 3′‐UTR microsatellite was instead highly polymorphic, and longer *Adcyap1* alleles were significantly associated with northern breeding latitudes, especially among males. The association between breeding latitude and *Adcyap1* polymorphism was considerably strong within the genetically homogenous cluster of long‐distance migrating northern birds, while it was nonsignificant within the cluster of short‐distance migrating southern birds.

Given the extremely low genetic polymorphism at *Clock*, we will frame our discussion around the *Adcyap1*–phenotype association.

### *Adcyap1* polymorphism and timing of migration

Relative timing of spring migration through the study site, accounting for phenological differences among populations, was not associated with *Adcyap1* 3′‐UTR microsatellite variation. We had no clear predictions on the association between *Adcyap1* polymorphism and timing of migration, as the few previous studies provided mixed or no evidence for links between *Adcyap1* and phenology (see Introduction). However, PACAP is broadly involved in scheduling circadian rhythms (Schwartz and Andrews 2013), and it may activate *Clock*, whose polymorphism covaries with breeding/migration phenology in some species (Liedvogel et al. 2009; Caprioli et al. 2012; Bazzi et al. 2015; Bourret and Garant 2015; Saino et al. 2015). The lack of any statistically detectable association between spring migration phenology and *Adcyap1* polymorphism may possibly indicate that phenology in this species is mostly shaped by other PCG, or by environmental modifications of gene expression via epigenetic mechanisms (Joska et al. 2014). Alternatively, phenology could be under the control of several genes with small additive effects (Manolio et al. 2009), as hypothesized for reproduction and other complex life‐history traits (Visser et al. 2010; Liedvogel et al. 2012).

### *Adcyap1* polymorphism and breeding destination

The observation of a relatively strong association between male *Adcyap1* polymorphism and breeding destination constitutes the first evidence of a link between interindividual variation in migration distance and PCG in wild birds. *Adcyap1* microsatellite polymorphism explained ca. 10% of the variance in male breeding destination, a remarkable value compared to studies analyzing genotype–phenotype associations in the wild (Fondon et al. 2008; Bourret and Garant 2015; Saino et al. 2015).

The association between *Adcyap1* allele size and breeding destination may be the outcome of both inter‐ and intrapopulation effects. For instance, *Adcyap1* may show a geographical cline, with northern birds having longer alleles than southern ones, but also, within populations, a pattern of individual birds bearing longer alleles migrating over longer distances. The observed difference in the strength of the association between allele size and feather *δ* ^2^H between the previously identified genetically homogeneous clusters of southern and northern birds suggests that within‐population rather than between‐population effects may drive the observed covariation between *Adcyap1* allele size and breeding destination in the entire sample. In line with this, although the two groups were genetically differentiated based on the *F*_ST_ statistic, the mean *Adcyap1* allele size did not significantly differ between northern and southern birds.

The remarkably strong association between *Adcyap1* polymorphism and breeding destination in long‐distance migrating northern birds (ca. 50% of variance explained; Table 2) compared to short‐distance migrating southern ones is intriguing. Previous evidence suggested that migratory behavior is under more strict endogenous (genetic) control in long‐distance vs. short‐distance migrants, because short‐distance migrants typically show larger phenotypic variance in migratory behavior than long‐distance migrants (Berthold 1996; Pulido and Widmer 2005). This has been hypothesized to result from strong stabilizing selection and environmental canalization of migratory traits (Pulido and Widmer 2005; Pulido 2007). Our findings suggest that a stronger endogenous control of migratory behavior in long‐distance migrants may also involve a tighter genotype–phenotype link for PCG. As these tests were conducted within previously identified genetically homogenous clusters (see Materials and Methods), the distinct spatial genetic structuring of the Wilson's warbler population complex did not confound our findings.

The association between *Adcyap1* genotype and breeding destination differed between the sexes. Using the entire sample of birds, *Adcyap1* allele size significantly predicted breeding destination in males but not in females. PCG may thus control migratory behavior in a sex‐specific way, as shown earlier for *Clock* and other genes (Caprioli et al. 2012; Bourret and Garant 2015). We could rule out that the lack of statistically significant genotype–phenotype association among females arose from the larger accidental subsampling of southern females. In fact, even when restricting the analysis to the northern birds (*n = *22), in spite of the low sample size (6 males and 16 females), the association between *Adcyap1* polymorphism and feather *δ* ^2^H was still stronger among males (*r *=* *−0.89) than females (*r *=* *−0.50). We could also rule out that feather *δ* ^2^H differently predicted actual breeding destination in the two sexes: the covariation between feather *δ* ^2^H and wing length was remarkably similar and statistically significant in both sexes (see also Q. R. Hays et al. in prep.).

Finally, we found that the effect of *Adcyap1* genotype on breeding latitude was consistent when we considered both the mean and the longer allele size, while no significant association emerged for the shorter allele size. This suggests a genetic dominance of the longer allele, a feature observed in the *Clock* gene (Liedvogel et al. 2009; Saino et al. 2015) and in other simple sequence repeats (Ross 2002; Fondon et al. 2008).

### Potential mechanisms driving the association between *Adcyap1* polymorphism and breeding destination

The mechanisms linking *Adcyap1* polymorphism to migration traits are largely unknown and have been only sparsely addressed (Mueller et al. 2011; Peterson et al. 2013; Saino et al. 2015). We envisage two nonexclusive pathways that may lead to an association between *Adcyap1* polymorphism and breeding destination among long‐distance migrating birds. These pathways rest on the assumption that the main determinant of migration distance in passerines is the amount and/or onset of migratory restlessness during their first southward migration (Gwinner 1996; Newton 2008).

First, a latitudinal cline of *Adcyap1* allele size may reflect adaptation to local photoperiodic conditions triggering migratory behavior from the highly seasonal northern breeding quarters at the most appropriate time of the year at different latitudes. In fact, PACAP is involved in conveying light information to the suprachiasmatic nucleus of the hypothalamus and modulates photoperiodic circadian genes (Hannibal et al. 1997; Nagy and Csernus 2007; Racz et al. 2008). *Adcyap1* length polymorphism may thus influence circadian genes' expression (Nagy and Csernus 2007; Racz et al. 2008) and regulate the onset of autumn migratory autumn restlessness. As the duration of autumn migratory restlessness mostly depends on the timing of its onset (Maggini and Bairlein 2010), *Adcyap1* could affect the onset rather than the duration of autumn restlessness in birds breeding at different latitudes. Unfortunately, the association between the onset of migratory restlessness and *Adcyap1* polymorphism was not tested in previous studies (Mueller et al. 2011; Peterson et al. 2013). Evidence from juvenile dispersing common buzzards may support this hypothesis. In this species, juveniles carrying longer *Adcyap1* alleles disperse earlier than those bearing shorter alleles (Chakarov et al. 2013). As both dispersal and migration rely on increased behavioral “restlessness” (Ritchison et al. 1992), we argue that *Adcyap1* polymorphism influences the onset of restlessness rather than its duration.

Second, PACAP may affect migratory restlessness, and hence, the distance to be migrated from different breeding latitudes in autumn, by modulating melatonin secretion (Simonneaux et al. 1993) and lipid utilization, while inhibiting feeding behavior (Tachibana et al. 2003, 2007). PACAP could cause a phase shift of the endogenous oscillators from day‐to‐night activity through melatonin modulation, a major determinant of migration onset in passerines (Fusani and Gwinner 2005; Mueller et al. 2011), and a fuel shift to lipid metabolism, which may influence nutritional state and night migratory activity (Coppack and Bairlein 2011; Schwartz and Andrews 2013). Longer *Adcyap1* alleles may induce a decrease of melatonin release over longer periods, extending migratory restlessness and thus the distance migrated (Coppack and Bairlein 2011).

### Concluding remarks

By taking advantage of feather *δ* ^2^H gradients throughout the breeding range of Wilson's warbler in western North America, we discovered the evidence of a very strong latitudinal cline in *Adcyap1* microsatellite polymorphism among the genetically homogeneous cluster of northern breeding birds, that were also genetically differentiated at this PCG from southern breeding ones. We also uncovered that the strength of *Adcyap1*–phenotype association may vary widely among geographical populations and even between the sexes, suggesting that sex‐specific selective pressures may affect PCG–phenotype associations. Importantly, between‐sex and among‐population variation in *Adcyap1–*phenotype associations may contribute to explain the often inconsistent findings of previous studies investigating the link between PCG polymorphism and migratory behavior.

On the whole, results from this study deepen our understanding of the genetic basis of avian migration, as they are consistent with the hypothesis that *Adcyap1* is involved in the regulation of migratory behavior, and may have far‐reaching evolutionary implications. For instance, our finding of stronger genetic differentiation for the *Adcyap1* microsatellite than for neutral microsatellite markers between short‐ and long‐distance migratory Wilson's warblers is coherent with a previous study comparing genome‐wide patterns of divergence between short‐ and long‐distance migrating populations of the Swainson's thrush (*Catharus ustulatus*) (Ruegg et al. 2014a). That study reported that several migration‐linked genes, including *Adcyap1*, showed a stronger pattern of single nucleotide polymorphism (SNP) differentiation between short‐ and long‐distance migrants compared to all the other SNPs from autosomal genes. Coupled with the observation of a strong latitudinal cline of allele size among long‐distance migrating northern birds, a significant differentiation of the *Adcyap1* microsatellite between short‐ and long‐distance migrating Wilson's warblers provides support for a role of migration‐linked genes and migratory behavior as drivers of population differentiation, promoting reproductive isolation and eventually leading to speciation (Liedvogel et al. 2011; Ruegg et al. 2014a).

## Conflict of Interest

None declared.

## Supporting information


**Appendix S1**. Determination of feather δ^2^H and genetic analyses.
**Appendix S2.** Comparison of *F*_ST_ of neutral microsatellites (from Clegg et al. 2003) between southern and northern Wilson's warblers.
**Table S1**. Wilson's warbler *Adcyap1* allele frequencies, observed heterozygosity (*H*_*o*_) and mean (SE) allele size.
**Table S2**. Phenotypic distribution of migration date of male and female Wilson's warblers homozygotes for *Clock*.
**Table S3**. Variation in the strength of the association between *Adcyap1* allele size and feather δ^2^H value among southern and northern Wilson's warblers based on different thresholds to identify southern and northern birds.
**Table S4.** Correlation between mean allele size at 8 neutral microsatellite markers (from Clegg et al. 2003) and breeding latitude or predicted feather δ^2^H of the breeding site.
