# Supplementary material for: Adcyap1 polymorphism covaries with breeding latitude in a Nearctic migratory songbird, the Wilson's warbler (Cardellina pusilla)
Source: Ecol Evol. 2016 Apr 7;6(10):3226–39. doi: 10.1002/ece3.2053 (PMC4870208; doi:10.1002/ece3.2053)
Supplement: Supplementary file 1 — Appendix S1. Determination of feather δ2H and genetic analyses. Appendix S2. Comparison of F ST of neutral microsatellites (from Clegg et al. 2003) between southern and northern Wilson's warblers. Table S1. Wilson's warbler Adcyap1 allele frequencies, observed heterozygosity (H o) and mean (SE) allele size. Table S2. Phenotypic distribution of migration date of male and female Wilson's warblers homozygotes for Clock. Table S3. Variation in the strength of the association between Adcyap1 allele size and feather δ2H value among southern and northern Wilson's warblers based on different thresholds to identify southern and northern birds. Table S4. Correlation between mean allele size at 8 neutral microsatellite markers (from Clegg et al. 2003) and breeding latitude or predicted feather δ2H of the breeding site. [file ECE3-6-3226-s001.doc]

**Supporting Information**

***Adcyap1* polymorphism covaries with breeding latitude in a Nearctic migratory songbird, the Wilson’s warbler (*Cardellina pusilla*)**

Gaia Bazzi, Andrea Galimberti, Quentin R. Hays, Ilaria Bruni, Jacopo G. Cecere, Luca Gianfranceschi, Keith A. Hobson, Yolanda E. Morbey, Nicola Saino, Christopher G. Guglielmo, Diego Rubolini

**List of items:**

**Appendix S1**. Determination of feather δ^2^H and genetic analyses.

**Appendix S2.** Comparison of *F*_ST_ of neutral microsatellites between southern and northern Wilson’s warblers.

**Table S1**. Wilson’s warbler *Adcyap1* allele frequencies, observed heterozygosity (*H_o_*) and mean (SE) allele size.

**Table S2**. Phenotypic distribution of migration date of male and female Wilson’s warblers homozygotes for *Clock*.

**Table S3**. Variation in the strength of the association between *Adcyap1* allele size and feather δ^2^H among southern and northern Wilson’s warblers based on different thresholds to identify southern and northern birds.

**Table S4.** Correlation between mean allele size at 8 neutral microsatellite markers and breeding latitude or predicted feather δ^2^H of the breeding site.

**Appendix S1.** Determination of feather δ^2^H and genetic analyses

*Determination of feather δ^2^H*

Tail feathers were first washed in a 2:1 chloroform/methanol solution to remove surface oils, rinsed and air dried. A small sample (ca. 0.35 mg) was cut from the distal vane of the feather and transferred to a silver capsule. In order to avoid uncontrolled exchange between non carbon-bound hydrogen in the feathers and ambient water vapor, δ^2^H analyses were performed using the comparative equilibration approach reported in . Feather and keratin standard δ^2^H values were analysed on H_2_ gases produced by flash pyrolysis under He flow. Within each feather run, we measured 5 replicates of the Environment Canada keratin reference materials CBS (-197‰), SPK (-121.6‰) and KHS (-54.1‰). Based on replicate measurements of standards, measurement error was estimated as ± 2‰.

*Genetic analyses*

DNA was extracted from feathers using the Dneasy® Blood & Tissue Kit (Qiagen, Hilden, Germany) following manufacturer’s instructions (i.e. Purification of Total DNA from Animal Tissues protocol), except for pre-treatment and elution steps. The basal portion (i.e., the portion of feather calamus from the tip to 2-3 mm above the superior umbilicus) of rectrices and breast contour feathers was isolated and incubated for at least six hours in sterile NaCl solution (0.9%) in agitation at 4°C. After rehydration, the calamus was chopped in small pieces on a sterile glass slide by using sterile instruments and incubated overnight in lysis buffer. After purification steps, DNA was eluted in 50 μl of deionized water (molecular biology grade) and DNA quality and concentration were measured fluorometrically with a NanoDrop™ 1000 Spectrophotometer (Thermo Scientific, USA).

Polymorphism at the *Clock* and *Adcyap1* genes (allele size due to the number of sequence repeats) were determined by fragment analysis as described in and , with some modifications. Differently from these two previous studies, given the lower DNA concentration of the collected samples, PCRs were conducted starting from 10 ng of DNA by using puReTaq Ready-To-Go PCR beads (Amersham Bioscience, Freiburg, Germany) in a 25 μl reaction according to the manufacturer’s instructions. Moreover, for both markers, the final elongation step (72°C) of PCR thermal profile was prolonged up to 30 minutes. PCR products (1 μl) were mixed with 12 μl of formamide and 0.2 μl of LIZ-500 size standard (Applied Biosystems, Foster City, CA, USA) and then analyzed on an ABI 3130 automated sequencer (Applied Biosystems).

The allele sizes for each locus were identified using Genemapper 4.0 software (Applied Biosystems). Genotyping (i.e. amplification and fragment electrophoresis) was repeated in samples with low or missing peaks at least two times before excluding them from the dataset.

**Appendix S2.** Comparison of *F*_ST_ of neutral microsatellites between southern and northern Wilson’s warblers.

We reanalysed microsatellite data previously published by to estimate *F*_ST_ at (presumably) neutral loci from breeding birds belonging to the same northern and southern population clusters of our study. We considered the data from the populations labelled JUN, WA and BC in Table 1 of as belonging to the northern population cluster (n = 42 individuals), whereas the population labelled CA was considered as belonging to the southern population cluster (n = 17 individuals). As in , for estimating *F*_ST_ we discarded data from three out eight microsatellites because of a significant heterozygote deficit. The estimated mean *F*_ST_ value across all 5 loci was -0.008 (95% bootstrap confidence intervals: -0.019 to 0.001) and not significantly different from 0 (*P* = 0.05). Hence, the *F*_ST_ point estimate for the *Adcyap1* microsatellite (0.045), besides being significantly different from 0, was considerably larger (outside the 95% confidence intervals) than the mean *F*_ST_ of the other five neutral microsatellites. Such a low level of genetic differentiation based on neutral microsatellites is in line with the lack of detectable divergence among western breeding populations of the Wilson’s warbler originally reported in . We note that significant patterns of genetic divergence among western breeding populations was later uncovered by by adopting a genomic approach.

*References*

Caprioli, M., Ambrosini, R., Boncoraglio, G., Gatti, E., Romano, A., Romano, M., Rubolini, D., Gianfranceschi, L. & Saino, N. (2012) *Clock* gene variation is associated with breeding phenology and maybe under directional selection in the migratory barn swallow. *PLoS ONE,* **7,** e35140.

Clegg, S.M., Kelly, J.F., Kimura, M. & Smith, T.B. (2003) Combining genetic markers and stable isotopes to reveal population connectivity and migration patterns in a Neotropical migrant, Wilson's warbler (*Wilsonia pusilla*). *Molecular Ecology,* **12,** 819-830.

Ruegg, K.C., Anderson, E.C., Paxton, K.L., Apkenas, V., Lao, S., Siegel, R.B., DeSante, D.F., Moore, F. & Smith, T.B. (2014) Mapping migration in a songbird using high-resolution genetic markers. *Molecular Ecology,* **23,** 5726-5739.

Saino, N., Bazzi, G., Gatti, E., Caprioli, M., Cecere, J.G., Possenti, C.D., Galimberti, A., Orioli, V., Bani, L., Rubolini, D., Gianfranceschi, L. & Spina, F. (2015) Polymorphism at the *Clock* gene predicts phenology of long-distance migration in birds. *Molecular Ecology,* **24,** 1758-1773.

Wassenaar, L.I. & Hobson, K.A. (2003) Comparative equilibration and online technique for determination of non-exchangeable hydrogen of keratins for use in animal migration studies. *Isotopes in Environmental and Health Studies,* **39,** 211-217.

**Table S1.** Wilson’s warbler *Adcyap1* allele frequencies, observed heterozygosity (*H_o_*) and mean (SE) allele size.

|  | 154 | 156 | 157 | 158 | 159 | 160 | 161 | 162 | 164 | *H_o_* | Mean  allele size |
| --- | --- | --- | --- | --- | --- | --- | --- | --- | --- | --- | --- |
| *Adcyap1* | 0.010 | 0.225 | 0.118 | 0.240 | 0.020 | 0.211 | 0.005 | 0.147 | 0.025 | 0.775 | 158.58 (0.16) |

**Table S2**. Phenotypic distribution of migration date of male and female Wilson’s warblers homozygotes for *Clock*. Migration date is expressed as residuals of a linear regression of migration date on δ^2^H, separately for each sex, in order to control for the effect of geographical origin on migration date (see Fig. 3). Mean values are shown together with SD (round brackets) and non-parametric bootstrap 95% confidence limits (see Methods) (square brackets). The values for the two heterozygote individuals are shown on the rightmost column: both values fall within the 95% confidence limits of the homozygous males and females, respectively.

|  | Homozygotes | Heterozygote |
| --- | --- | --- |
|  |  |  |
| Males (n = 55) | 0.01 (12.83) [-2.98 to 3.74] | -0.30 |
| Females (n = 45) | 0.05 (12.12) [-3.68 to 3.43] | -2.31 |

**Table S3**. Variation in the strength of the association between *Adcyap1* allele size and feather δ^2^H among southern and northern Wilson’s warblers based on different thresholds to identify southern (S) and northern birds (N) [N: feather δ^2^H < -130 ‰ or < -140 ‰; S: feather δ^2^H ≥ -80 ‰ or ≥ -90 ‰; see Methods for details]. Numbers in parentheses denote sample sizes. Pairs of letters highlight statistically significant (*P* < 0.01) Z-tests of the difference between correlation coefficients of southern and northern birds, with letters indicating different correlations (superscripts). Significance levels of correlation coefficients: * = *P* < 0.05; ** = *P* < 0.01; *** = *P* < 0.001.

|  | S (≥ -80 ‰)^a^ | S (≥ -90 ‰)^b^ | N (< -130 ‰)^c^ | N (< -140 ‰)^d^ | Z-test |
| --- | --- | --- | --- | --- | --- |
|  |  |  |  |  |  |
| Mean allele size | 0.07 (35) | -0.12 (43) | -0.69 (22)*** | -0.71 (17)** | ac-ad-bc-bd |
| Short allele size | -0.05 (35) | -0.13 (43) | -0.31 (22) | -0.40 (17) |  |
| Long allele size | 0.05 (35) | -0.07 (43) | -0.77 (22)*** | -0.73 (17)** | ac-ad-bc-bd |
|  |  |  |  |  |  |

**Table S4**. Correlation between mean allele size at 8 neutral microsatellite markers and breeding latitude or predicted feather δ^2^H of the breeding site. Microsatellite data refer to the breeding populations labeled JUN, WA and BC in Table 1 of . These populations occur in the latitude range spanning between 48°N and 58°N, corresponding to predicted feather δ^2^H spanning between -123 ‰ and -157 ‰ based on our feather isoscape (see Fig. 1). Hence, the range of these three populations entirely encompasses the ‘northern breeding populations’ as defined in this study (δ^2^H < -130 ‰; see Methods). As the original microsatellite data from these three populations were not matched to individual-based isotope data , for each breeding population we calculated the predicted mean feather δ^2^H from a buffer of 50 km around each breeding site from the raster of the feather isoscape depicted in Fig. 1. Correlation coefficients and sample size (number of individuals; in parentheses) are shown. None of the correlations is statistically significant (all *P* > 0.09).

| Locus | Latitude | Feather δ^2^H |
| --- | --- | --- |
| *Wp*C6 | 0.120 (41) | -0.127 (41) |
| *Wp*D23 | 0.055 (42) | -0.052 (42) |
| *Wp*D30 | 0.133 (41) | -0.126 (41) |
| *Wp*D4 | 0.248 (42) | -0.240 (42) |
| *Dpμ*01 | 0.212 (41) | -0.198 (41) |
| *Dpμ*03 | -0.085 (42) | 0.084 (42) |
| *Dpμ*05 | -0.119 (39) | 0.121 (39) |
| *Dpμ*16 | -0.257 (41) | 0.265 (41) |
